# Microclimate drives demographic compensation in a narrow endemic tropical species

**DOI:** 10.1111/nph.70944

**Published:** 2026-02-11

**Authors:** Talita Zupo, Diego Fernando Escobar, Gabriel S. Santos, Vitor de Andrade Kamimura, Yan Nunes Dias, Rafael L. de Assis, Cecílio F. Caldeira, Maurício Takashi Coutinho Watanabe, Rita de Cássia Quitete Portela, Valeria Tavares, Carolina da Silva Carvalho

**Affiliations:** ^1^ Instituto Tecnológico Vale Rua Boaventura da Silva 955 Belém CEP 66055‐090 Pará Brazil; ^2^ Departamento de Ciências Biológicas Universidade Federal do Espírito Santo (UFES) Rua Fernando Ferrari, 514 Vitória CEP 29075‐910 Espírito Santo Brazil; ^3^ Departamento de Biologia Vegetal Universidade Estadual de Campinas (UNICAMP) Monteiro Lobato st., 255 ‐ Cidade Universitária Campinas 13083‐862 São Paulo Brazil; ^4^ Departamento de Ecologia, Instituto de Biologia Universidade Federal do Rio de Janeiro (UFRJ) Rua Antônio Barros de Castro, 119 Rio de Janeiro CEP 21941‐853 Rio de Janeiro Brazil; ^5^ Museu Paraense Emílio Goeldi Programa de Pós‐graduação em Biodiversidade e Evolução Av. Perimetral, 1901 Belém CEP 66077‐830 Pará Brazil; ^6^ Departamento de Sistemática e Ecologia Universidade Federal da Paraíba (UFPB), Programa de Pós‐Graduação em Ciências Biológicas Campus I, Castelo Branco João Pessoa CEP 58051‐900 Paraíba Brazil

**Keywords:** Amazon canga (*Campo rupestre*), environmental heterogeneity, matrix population models, plant demography, vital rates

## Abstract

- Demographic compensation occurs when reductions in some vital rates are offset by increases in others, allowing populations to maintain similar performance across varying environments. This mechanism may help explain species' ecological distributions and range limits, yet its role at microenvironmental scales remains poorly understood. We investigated demographic compensation in *Ipomoea cavalcantei*, a narrow‐range but locally abundant species endemic to Amazonian ironstone outcrops, by comparing populations in two contrasting habitats: open‐ and shrubby‐canga.
- Using 3 yr of demographic data, we built matrix population models and conducted a life table response experiment. We also carried out germination and seedling establishment experiments under different temperature and light conditions simulating both habitats to identify the potential environmental drivers and their effects on key life‐cycle events.
- Despite contrasting environmental conditions, both populations exhibited similar population growth rates (λ), with opposing contributions of growth and fecundity – evidence of demographic compensation. The open‐canga population had lower growth but higher recruitment, driven by favorable temperature regimes for seed dormancy release and germination. Reduced growth was associated with physiological stress under high irradiance and shallow soils.
- Our results show that demographic compensation allows *I. cavalcantei* to persist across microhabitats, highlighting the importance of fine‐scale environmental heterogeneity in shaping species distributions.

## Introduction

Understanding the biotic and abiotic factors that influence species persistence and range limits, particularly for species with restricted distributions, is crucial to predicting species' long‐term viability. Several hypotheses have been proposed to explain species range limits, among them the center‐periphery hypothesis, which posits that populations exhibit reduced demographic performance (population growth rate and abundance) the farther they are from their optimal environmental conditions. Under suboptimal conditions, growth, survival, and fecundity (vital rates) decrease, ultimately limiting species occurrence (Gaston, 2009; Abeli *et al*., 2014; Pironon *et al*., 2017). Nevertheless, this hypothesis has not been supported by several organisms (Abeli *et al*., 2014; Pironon *et al*., 2017), as vital rates vary across space and time due to differential effects of environmental factors throughout a species' distribution range (Ehrlén *et al*., 2016; Pironon *et al*., 2018). In other words, optimal conditions for one vital rate are not necessarily optimal for all vital rates.

Recent studies have found evidence that species can compensate for reductions in some vital rates by others that co‐vary in opposite directions, resulting in populations that can attain similar performance, a phenomenon called demographic compensation (Doak & Morris, 2010; Villellas *et al*., 2015). In this sense, demographic compensation provides a mechanism for population persistence, enabling species to modulate their dynamics in response to environmental factors. As a result, demographic compensation has been suggested as a mechanism that allows species to expand their geographical range size or to occupy a greater variety of habitats within their range, which is not expected in the absence of demographic compensation (Doak & Morris, 2010; Villellas *et al*., 2015; Yang *et al*., 2022). Hence, demographic compensation may be crucial for understanding species' current distribution and, therefore, accurately predicting range shifts in response to ongoing environmental changes (Villellas *et al*., 2015). Indeed, Villellas *et al*. (2015) analyzed demographic compensation in 22 species and found that it likely increased the size of a species' geographical range in comparison to when all vital rates respond similarly to environmental gradients, due to the reduction of the spatial variation in population growth rates. In addition, they found that fecundity (reproduction and recruitment) was the vital rate most frequently involved in demographic compensation. Villellas *et al*.'s (2015) seminal work not only provided some of the first empirical evidence across multiple species but also helped to establish demographic compensation as a potentially widespread phenomenon shaping species' range.

Unfortunately, broad conclusions about the prevalence of demographic compensation across species cannot be reached, given the strong geographic bias in available demographic data and the limited understanding of how negative correlations among vital rates (e.g. between survival and fecundity) can stabilize population growth rates under different environmental conditions along species' range. Addressing this limitation requires long‐term demographic studies across multiple populations to accurately estimate demographic parameters and link variation in vital rates and population growth rates to abiotic and biotic drivers (Caswell, 2000; Ehrlén *et al*., 2016). Yet, the few existing studies are conducted in temperate zones (Sheth & Angert, 2018; Yang *et al*., 2022; but see Souza *et al*., 2018), with species that have wide geographic distributions, usually along a latitudinal (Doak & Morris, 2010; Villellas *et al*., 2013) or altitudinal gradient (García‐Camacho *et al*., 2012). At these scales, climate‐vital rate relationships are usually inferred from correlations that use coarse climatic data (regional), overlooking the need for fine‐scale (local) climate data when examining effects on individual performance and population dynamics (Ehrlén *et al*., 2016; Griffith *et al*., 2016; Christiansen *et al*., 2024). Importantly, microclimate can be key to understanding variation in plant performance, particularly in highly heterogeneous environments and in narrow endemic species (Oldfather & Ackerly, 2019; Christiansen *et al*., 2024; Kemppinen *et al*., 2024). Additionally, purely correlative approaches make it difficult to establish causal relationships; however, experiments aimed at uncovering the mechanisms underlying demographic compensation are rarely conducted (but see DeMarche *et al*., 2018; Oldfather *et al*., 2021). Thus, incorporating experimental manipulations can be especially valuable for identifying potential environmental drivers and their effects on key life‐cycle events (Ehrlén *et al*., 2016).

The combination of edaphic conditions and the diverse geomorphological mosaic of the Amazonian *cangas* creates multiple microhabitats that vary in temperature, light, and water availability (Jacobi *et al*., 2007; Giulietti *et al*., 2019), providing an ideal setting to test how microclimatic variation can drive demographic compensation in heterogeneous environments. *Cangas* are a specific type of *campo rupestre* found on ironstone outcrops characterized as a fire‐prone seasonally dry grassy‐shrubby vegetation mosaic with shallow, acidic, nutrient‐depleted, and iron‐rich soils (Silveira *et al*., 2016; Miola *et al*., 2021). In addition to the nutrient‐poor soils, *canga* ecosystems are marked by high solar radiation, hot temperatures, low water retention capacity, strong winds, and a severe drought period (Jacobi *et al*., 2007; Schaefer *et al*., 2016a), subjecting vegetation to harsh yet seasonal environmental conditions (Le Stradic *et al*., 2015; Oliveira *et al*., 2015; Silveira *et al*., 2016). These unique conditions contribute to the remarkable richness of endemic plant species found in cangas (Giulietti *et al*., 2019). Considering all *canga* habitats, the open rupestrian vegetation (locally known as *canga couraçada* and referred to as open‐canga henceforth) and the shrubby rupestrian vegetation (referred to as shrubby‐canga henceforth) are amongst the most representative. While the shrubby‐cangas have deeper soils that support a dominant shrubby layer with plants reaching up to 4 m, open‐cangas have very shallow soils (*c*. 5 cm deep) and are dominated by herbaceous species (Mota *et al*., 2015; Viana *et al*., 2016), which experience higher radiation, light incidence, and temperatures at the soil level, potentially categorizing them as a harsher environment.

Here, we analyzed the population dynamics of a narrow and locally abundant endemic species, *Ipomoea cavalcantei* (Convolvulaceae), which occurs in highly heterogeneous habitats. Specifically, we studied *I. cavalcantei* populations occurring in the open‐canga and in the shrubby‐canga to test whether demographic compensation could explain the species' ecological distribution by expanding the range of environments over which populations can succeed, thus buffering populations from spatial environmental variation. If demographic compensation is present, both populations will show similar growth rates but different relative vital rate contributions. If absent, we expect the open‐canga population (subjected to harsher environmental conditions than the shrubby‐canga population) to show a lower population growth rate, resulting from lower vital rate values for survival, growth, and fecundity. In addition, given that demographic compensation often occurs between survival or growth and fecundity (Villellas *et al*., 2015; Yang *et al*., 2022), and fecundity encompasses many regeneration processes (Larson & Funk, 2016), we also examined how microclimatic conditions in both vegetation types affect several regeneration processes by combining both field and experimental data. Specifically, we analyzed seed production, seedling emergence, and seedling mortality in the field, carried out seed germination experiments under different alternating temperatures, and conducted a seedling establishment experiment, where seedlings were grown under two light conditions simulating the vegetation types in which they occur. By integrating demographic data with manipulative experiments, our study provides a stronger framework to uncover the mechanisms underlying demographic compensation and spatial demographic variation. This approach enables us to establish causal links between vital rates and their environmental drivers, thereby improving our understanding of how demographic compensation operates at microenvironmental scales.

## Materials and Methods

### Study site and species

**Fig. 1:**
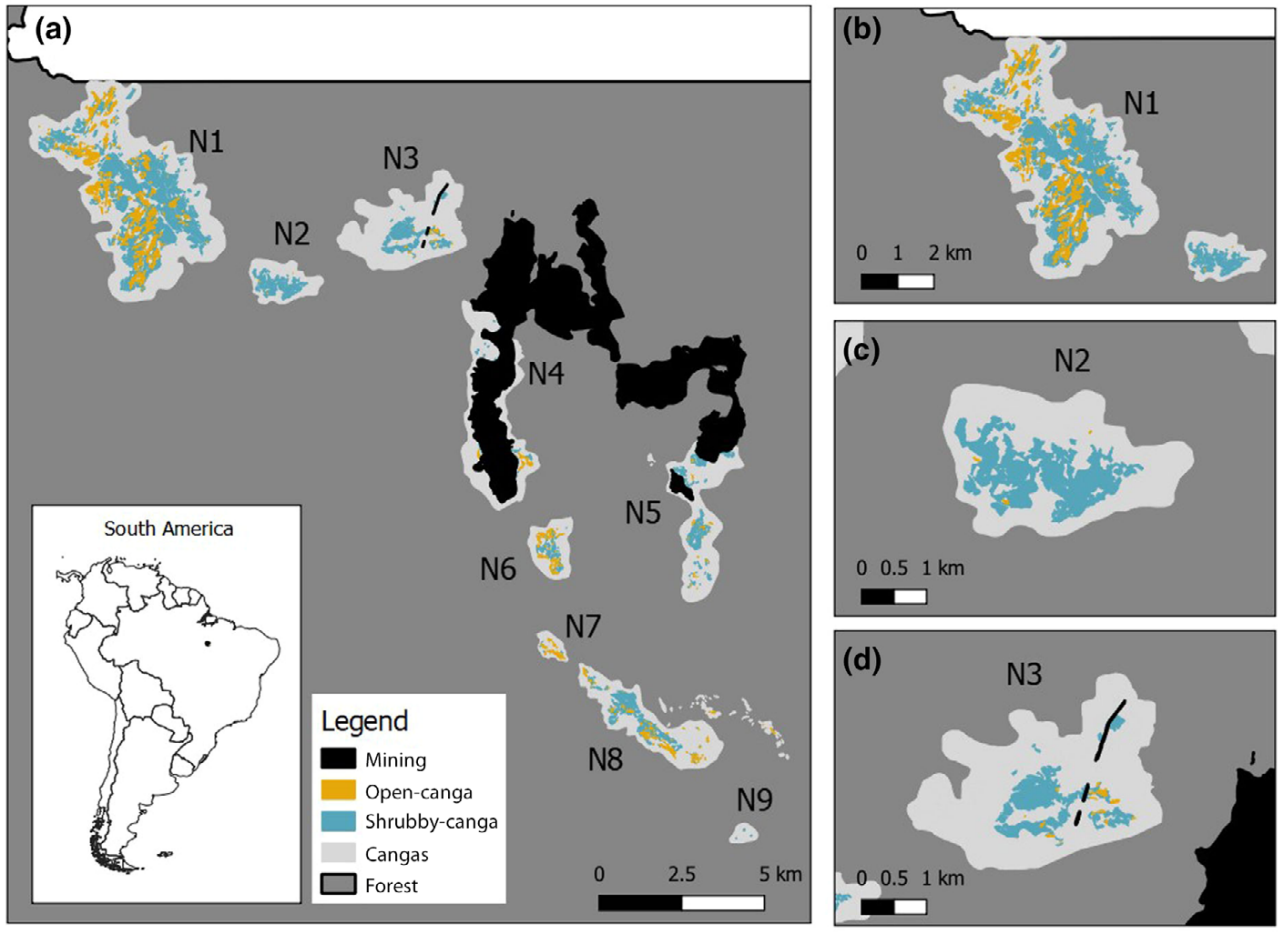
Geographic distribution of canga patches and natural populations of *Ipomoea cavalcantei*. (a) Distribution of the *canga* patches of the northern mountain range (*Serra Norte*) of the FLONA Carajás, north of Brazil. *Ipomoea cavalcantei* naturally occurs in patches N1‐ N5, mainly in open‐ and shrubby‐cangas. Currently, populations of *I. cavalcantei* not impacted by mining are only found throughout N1 (b), N2 (c), and N3 (d) patches, where the amount of open‐ and shrubby‐cangas differ.

The study was carried out in the Carajás National Forest (FLONA Carajás), located in the eastern Amazon (Pará State, Brazil). This region is characterized by vast forests surrounding iron‐rich mountain tops, where the *canga* ecosystems occur. The *canga* ecosystems in the FLONA Carajás are distributed within two areas: the northern mountain range (locally known as *Serra Norte*), in which *canga* occurs in patches or plateaus of different sizes, referred to as N1 to N9 (Fig. 1a), and the southern mountain range (locally known as *Serra Sul*), where a large continuous *canga* patch is found (S11, *c*. 48.1 km^2^), together with other smaller patches (Souza‐Filho *et al*., 2019). The climate is hot and humid with a marked dry season from May to October and a rainy season from November to April, where most of the annual precipitation occurs (1800–2300 mm; Viana *et al*., 2016).

In *canga* ecosystems, patterns of species composition and distribution are highly linked to edaphic factors, where contrasting vegetation types occur just a few meters away from each other (Mota *et al*., 2015; Schaefer *et al*., 2016a). *Ipomoea cavalcantei* D.F. Austin is only found in five *canga* plateaus from the northern mountain range (N1 to N5) that collectively measure *c*. 20 km^2^ (Babiychuk *et al*., 2019; Fig. 1a). However, mining activities are ongoing in two patches (N4 and N5), and thus, in these areas, the species' natural habitat is almost entirely depleted.

*Ipomoea cavalcantei* is a perennial twining shrub with woody stems and enlarged storage roots that occurs mainly throughout the open‐ and shrubby‐canga vegetation types (Babiychuk *et al*., 2019; Rodrigues *et al*., 2020; Fig. 1b–d). In shrubby‐cangas, *I. cavalcantei* frequently exhibits twining behavior with shoots reaching the canopy (4–6 m high), whereas in open‐cangas, plant shoots are short and erect (Babiychuk *et al*., 2019). Additionally, *I. cavalcantei* is a self‐incompatible species and hummingbirds are the main pollinators (Babiychuk *et al*., 2019). Seeds are wind‐dispersed between May and July, and, as in other Convolvulaceae, seeds are likely to display physical seed dormancy (Jayasuriya *et al*., 2008).

**Fig. 2:**
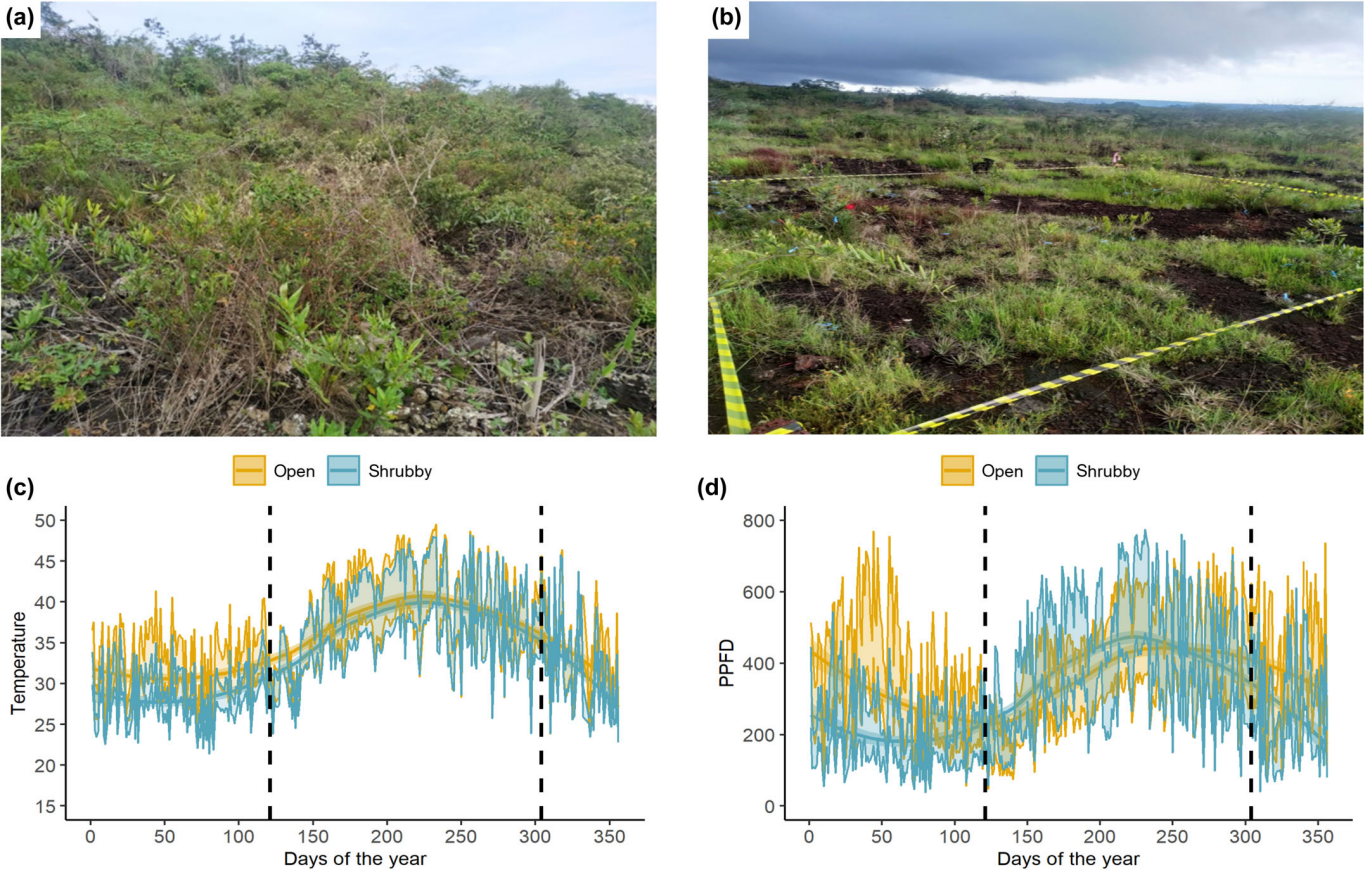
Environmental characterization of open‐ and shrubby‐canga habitats in FLONA Carajás. (a, b) Photographs of the study sites showing shrubby‐canga (a) and open‐canga (b). (c) Daily daytime (06:00–18:00 h) temperatures (mean ± SE) and (d) photosynthetic photon flux density (PPFD; mean ± SE) at the soil level in the open‐cangas (yellow) and shrubby‐cangas (blue) of the FLONA Carajás. Dashed vertical lines represent the beginning (day 121; May) and end (day 304; October) of the dry season.

To characterize the microclimatic conditions of both vegetation types, we registered temperature and light incidence at the soil level. For this reason, we placed 3–4 temperature and light sensors (Hobo MX2202) in each vegetation type; sensors were placed on the soil level to record temperature and light as perceived by a seed and a seedling. Sensors recorded temperature (°C) and solar light intensity (lux) every hour for 1 yr (December 2022 to December 2023; Fig. 2). Lux was transformed into photosynthetic photon flux density (PPFD) using the lux‐PPFD conversion factor for sunlight (0.0185; Thimijan & Heins, 1983).

### Demographic parameters and plant performance across vegetation types

#### Experimental design and stage classification

We established permanent plots of 25 m^2^ (5 × 5 m) along the two *canga* vegetation types of the N1 plateau: five plots in the open‐canga (a total of 179 individuals tagged in 2022) and six plots in the shrubby‐canga (a total of 102 individuals tagged in 2022). In each plot, all individuals of *I. cavalcantei* (including seedlings) were identified with a metal tag and measured. For every individual, we recorded the total number of shoots (accounting for vegetative and reproductive shoots), height, length of the longest shoot, and life stage. We established three stages: seedlings – distinguished by the presence of cotyledons; nonreproductive (immature) individuals – devoid of buds, flowers, and fruits; and reproductive (mature) individuals – exhibiting buds, flowers, and/or fruits. Annual censuses were conducted during the species' flowering peak (March/April) in 2022, 2023, and 2024.

#### Seed production and fecundity

To estimate seed production of each individual within plots, we first sampled 20 individuals outside the plots in each vegetation type. For these 20 sampled individuals, we counted the number of both vegetative and reproductive shoots, and for each reproductive shoot, we counted the number of reproductive structures (buds, flowers, and fruits). Sampling was conducted in April 2022, 2023, and 2024. The mean number of fruits per reproductive shoot was estimated for each vegetation type as:

$$\text{Mean fruits}\;\mathrm{per}\;\text{shoot}=\begin{array}{l} \text{number of fruits}\\ +\left(\text{number of flowers and buds}\times 0.12\right), \end{array}$$

where 0.12 represents the proportion of flowers that successfully develop into fruits (Valentin‐Silva *et al*., 2025).

Given that each fruit produces four seeds and that 70% of seeds are predated before dispersal (Supporting Information Methods S1 – Estimation of seed predation before dispersal), the mean number of seeds produced per reproductive shoot was estimated as:

$$\begin{array}{l} \text{Viable seeds}\;\mathrm{per}\;\text{reproductive shoot}\\ \;=\text{mean fruits}\;\mathrm{per}\;\text{shoot}\times 4\times 0.3. \end{array}$$

For individuals within the plots, we recorded the number of reproductive shoots per individual. The total number of viable seeds produced by each individual was then calculated by multiplying the number of reproductive shoots by the mean number of viable seeds per reproductive shoot derived from the individuals sampled outside the plots.

With these estimates, we calculated fecundity (*f*_i_) for individuals within the plots as the number of seedlings at *t* + 1 divided by the number of viable seeds produced per reproductive individual at *t*, with *t* being the annual census. Seedling number was defined as the total number of seedlings recorded in each plot at the annual census. Fecundity was assigned to individual plants as these varied in the number of reproductive shoots (and thus resulted in different seed production).

With the mean number of fruits per reproductive shoot estimated for individuals within plots, we also calculated the mean number of fruits (and thus viable seeds) produced per m^2^ in each vegetation type in each year (Seed production m^−2^).

#### Construction of projection matrices

**Fig. 3:**
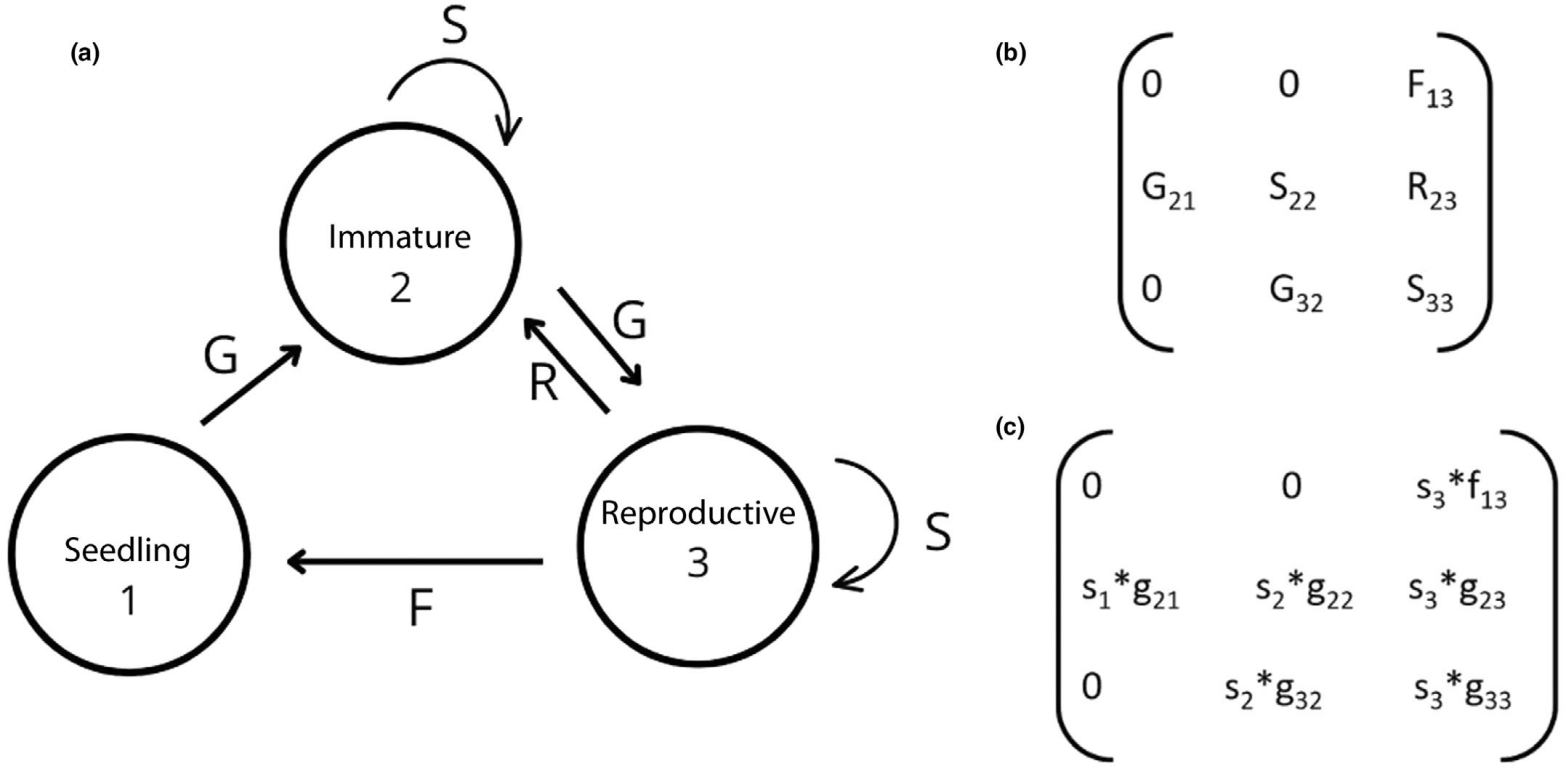
Life cycle and demographic parameterization of *Ipomoea cavalcantei* used in the population projection model. (a) Life cycle graph showing the stage structure of *I. cavalcantei*, where circles represent stages and arrows indicate all possible transitions between stages. (b) Population projection matrix corresponding to the life cycle graph in (a), with elements defined as upper‐level vital rates. (c) Decomposition of upper‐level vital rates into lower‐level vital rates. Stasis (*S*_*ij*_) is the probability of survival and remaining in the same stage expressed as *s*_i_*g_*ij*_ (e.g. S_22_ = s_2_*g_22_); Growth (*G*_*ij*_) is the probability of survival and growth to the next stage expressed as *s*_i_*g_*ij*_ (e.g. G_32_ = s_2_*g_32_); Regression (*R*_*ij*_) is the probability of survival and regression to a previous stage expressed as *s*_i_*g_*ij*_ (R_23_ = s_3_*g_23_); and fecundity (*F*_*ij*_), which is also contingent on survival (*s*_i_*f_*i*_). *s*_*i*_, *g*_*i*_, and *f*_*i*_ represent the lower‐level vital rates: survival, growth, and reproduction, respectively. In this model, we treated all transition intervals as annual.

We constructed two Lefkovitch matrices for each site using demographic parameters obtained from the data collected during each annual transition interval (Caswell, 2006). The projection matrices derived from the life cycle of *I. cavalcantei* (Fig. 3a), including all possible transitions between ontogenetic stages observed in the study model, resulted in a 3 × 3 matrix (Fig. 3b). The projection matrix is composed of matrix elements (*a*_*ij*_) that represent the transition probabilities or fecundity rates, describing how stage *j* at time *t* contributes to stage *i* at time *t* + 1 (Caswell, 2001; Figs 3b, S1, S2). Details on how the matrix was constructed are provided in Figure B and in the Methods S1 – *Construction of projection matrices*. For all matrix analyses, we used the popbio package (Stubben & Milligan, 2007) in R (R Development Core Team, 2024). Then, we calculated the asymptotic population growth rate (λ; dominant eigenvalue) using each transition matrix (Caswell, 2001). To verify differences in λ between sites, we calculated bias‐corrected 95% confidence intervals (CIs) for each λ by bootstrapping using the ‘boot.transitions’ function of the popbio package (Stubben & Milligan, 2007). We constructed 2000 bootstrapped matrices by randomly sampling individuals, with replacements from the data for each stage, maintaining the same number of observations at each time interval. The λ values of the 2000 replications were then averaged and the 95% CIs were calculated using the percentiles of the distribution (Caswell, 2001).

To explore how proportional changes in vital rates (elasticity) could affect the population growth rate (λ; Caswell, 2001), we carried out prospective analyses for which we used the *eigen.analysis* function of the popbio package with the mean matrix for each vegetation type (Stubben & Milligan, 2007).

#### Life table response experiments

To test if there was evidence of demographic compensation, we used a Life Table Response Experiment (LTRE) to quantify the contribution of vital rates to the differences in λ observed between both populations (Horvitz *et al*., 1997; Villellas *et al*., 2015). We used a fixed‐design experiment with the mean matrix (of both years) for each vegetation type; the shrubby‐canga population of *I. cavalcantei* was used as the reference population (Horvitz *et al*., 1997; Caswell, 2006). Contributions of matrix elements corresponding to growth and stasis were summed to represent the overall contribution of growth and stasis, respectively, because we were interested in the vital rates driving the observed differences in λ rather than stage‐specific contributions.

To quantify uncertainty in the LTRE contributions, we used the same sets of bootstrapped transition matrices described above. For each LTRE bootstrap iteration, we randomly sampled one bootstrapped matrix from each year within a habitat and averaged these two matrices to obtain a habitat‐level transition matrix for that iteration. This procedure was repeated independently for the open‐ and shrubby‐canga populations, producing paired habitat‐level matrices for each bootstrap replicate. We then applied the fixed‐design LTRE to each bootstrap pair to estimate the contributions of growth, stasis, regression, and fecundity to differences in λ between habitats. The mean contributions and 95% confidence intervals were then derived from 1000 bootstrap replicates. All analyses were carried out with the *classicalLTRE* function from the exactLTRE package in R (Hernández *et al*., 2023).

#### Population performance in open and shrubby cangas

To test whether stage structure differs between vegetation types (open‐ and shrubby‐canga), we analyzed the density of each ontogenetic stage (number of individuals/m^2^), seed production, seedling emergence (number of seedlings m^−2^), and survival after the first dry season, with generalized linear models (GLMs). We fitted an additive model with site and year as fixed factors. For the density of each ontogenetic stage, we used quasipoisson distribution and log‐link function. The number of seeds m^−2^ was log‐transformed and fitted with a Gaussian distribution and identity link function, and the probability of seedling survival fitted with binomial distribution and logit link (Zuur *et al*., 2009).

### Experimental response of seed germination, seed dormancy release, and seedling establishment to abiotic factors

#### Seed germination experiments

We conducted germination experiments under different alternating temperatures to evaluate the effect of temperature on seed germination of *I. cavalcantei*. These experiments allowed us to understand if soil temperatures act as a filter for seed germination, potentially driving differences in plant performance between open‐ and shrubby‐cangas. Seeds collected from multiple maternal plants were exposed to four different alternating temperatures (night/day: 20/25, 20/30, 20/35, and 20/40°C; 12 h under each temperature) and monitored for 20 d. The temperature treatments were based on soil temperatures measured during the rainy season in open‐ and shrubby‐cangas, which exhibit different daily temperature cycles (20/35 and 20/30°C, respectively). Although the mean daily temperatures differed only slightly between habitats (28 ± 2.3°C in open‐cangas and 26.1 ± 1.8°C in shrubby‐cangas), these correspond to the 20/35 (27.5°C) and 20/30 (25°C) daily regimens. We also included cooler (20/25°C) and warmer (20/40°C) daily cycles to broaden the range of diurnal fluctuations (5–20°C) and mean temperatures (22.5–30°C) observed across canga vegetation types.

We quantified seed germination and viability under alternating temperatures, and subsequently estimated germination thresholds using time‐to‐event curves and thermal‐time models. These analyses estimate the base and ceiling temperatures for germination, allowing us to determine whether temperature differences between vegetation types can drive contrasting early‐life performance. Full methodological procedures, statistical modelling approaches, and model comparisons are provided in Methods S1 – *Seed germination experiments*.

#### Dormancy release and viability

Because seed dormancy can influence recruitment patterns and buffer populations against temporal variation, and many Convolvulaceae produce physically dormant seeds (Jayasuriya *et al*., 2008), we tested whether seeds exhibit physical dormancy and assessed the effect of alternating temperatures (representative of canga microhabitats) on dormancy release and viability. Dormancy status of recently dispersed seeds (baseline of dormancy proportion) and of seeds subjected to alternating temperature treatments was inferred based on imbibition, germination, tetrazolium testing, and mechanical scarification (Table S1). By linking seed dormancy to germination patterns, these experiments provide critical context to interpret recruitment in the field. Comprehensive methodological details, model specifications, and analytical procedures are available in Methods S1 – *Dormancy release and viability*.

#### Seedling establishment

To determine if differences in light incidence affect seedling growth, we evaluated above and belowground biomass and the efficiency of the photosystem II. To do so, we carried out a potted experiment where seedlings were set to grow under two light conditions: full sunlight and 70% sunlight, with a total of 84 seedlings (42 per treatment). The seedlings were obtained from the germination experiments detailed above and the experiment was carried out in a glasshouse located in Belém city, Pará state, Brazil. The 70% sunlight condition was simulated with a mesh that blocked 30% of the irradiance. These light conditions were established based on light incidence data registered in both vegetation types, mimicking the light conditions of open‐ and shrubby‐cangas, respectively. Our light sensors recorded a mean PFFD *c*. 30% less in shrubby‐cangas in relation to open‐cangas (Fig. 2; Table S2). The experiment was carried out for 6 months to coincide with the maximum period a seed has to germinate, establish, and grow before the dry season in canga ecosystems. More details on the seedling establishment experiment can be found in the Methods S1 *Seedling establishment experiment*.

Every month, seven individuals of each light condition were randomly selected, harvested, and measured. Before harvesting, we measured the quantum efficiency of PSII ($F_{v}^{\prime }/F_{m}^{\prime }$) in fully expanded leaves (three measurements per plant) with a MultispeQ portable fluorometer (PhotosyngQ, East Lansing, MI, USA) and recorded if cotyledons were still present. We then harvested the plants and took them to the laboratory, where the soil was carefully washed from the roots, and their biomass was weighed. The fresh and dry weight of both above‐ and belowground biomass was weighed separately. Moreover, the belowground biomass was further separated into fine roots and bud‐bearing storage systems – as these represent the investment in absorption and storage, respectively – and also weighed separately. After determining the fresh weight, the specific plant parts were properly identified, put in paper bags, dried at 70°C for 72 h, and weighed with an analytical balance (0.0001 g) to a constant dry weight.

To evaluate the light effects on seedling growth, we used a GLM for each response variable: aboveground biomass, belowground biomass (fine roots + storage organ), fine root biomass, storage organ biomass, root : shoot ratio, fine root: storage ratio, and quantum yield ($F_{v}^{\prime }/F_{m}^{\prime }$). Biomass response variables were log transformed, and GLMs were fitted with a Gaussian distribution and identity link function with treatment and time as fixed factors; quantum yield was fitted with a beta distribution and logit link function with treatment and time as fixed factors (Zuur *et al*., 2009).

## Results

### Demographic parameters

#### Asymptotic population growth rate (λ)

**Fig. 4:**
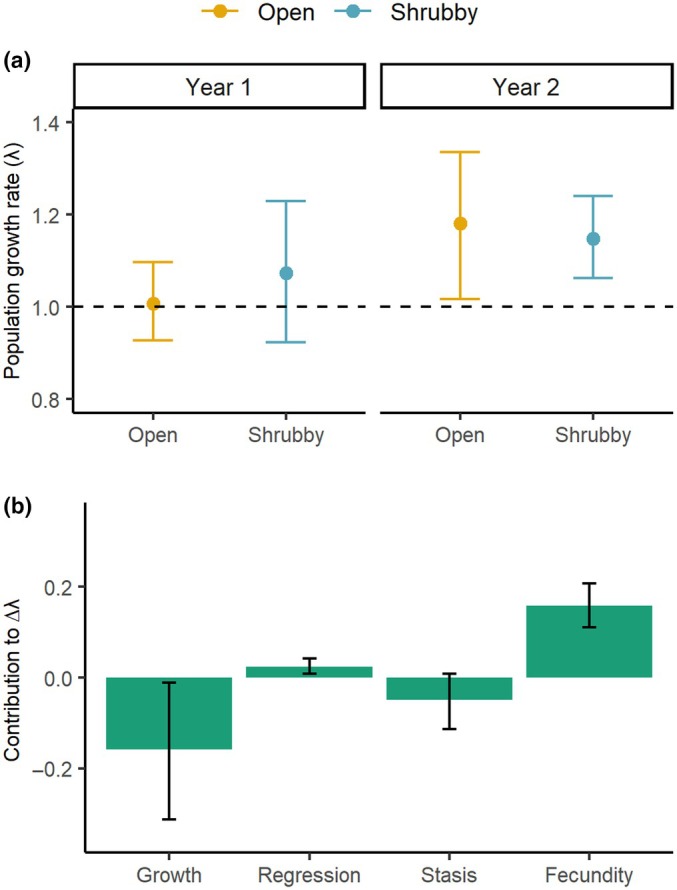
Population growth rates and retrospective (LTRE) analysis of population growth differences between open‐ and shrubby‐canga populations of *Ipomoea cavalcantei*. (a) Asymptotic population growth rates (λ) for the populations of *I. cavalcantei* in open‐canga (yellow) and shrubby‐canga (blue) vegetation types. Each point represents the mean, and the error bars represent the 95% CIs after bootstrapping each annual transition matrix. Year 1 refers to the 2022–2023 transition and year 2 to the 2023–2024 transition. (b) Vital rate (growth, regression, stasis, and fecundity) contributions to the observed differences in population growth rates (Δλ) between the open‐ and shrubby‐canga populations of *I. cavalcantei*. The LTRE was performed with the mean matrix calculated for each population, and the error bars represent the 95% CIs; we summed the contribution of growth and stasis of all stages to represent overall growth and stasis, respectively. The shrubby‐canga population was used as the reference population.

The average asymptotic population growth rate (λ) of *Ipomoea cavalcantei* was similar between populations in both years, with the *CIs* overlapping throughout the study period (Fig. 4a). Both populations were essentially stable (λ ~ 1) during the first transition interval (2022–2023), but λ increased for both in the second transition (2023–2024). In open‐canga sites, λ rose from 1.01 to 1.18, while in shrubby‐canga sites it increased from 1.09 to 1.15, with a similar Δλ between populations—0.08 in the first transition and 0.03 in the second (Fig. 4a).

#### LTRE analysis

Despite similar population growth rates (λ), LTRE analysis revealed opposite vital‐rate contributions between populations (Fig. 4b), indicating demographic compensation. Growth and fecundity were the primary drivers: open‐canga individuals exhibited reduced growth but greater fecundity relative to shrubby‐canga individuals. This compensation pattern persisted across years but intensified in the second transition, when the negative contributions of growth and stasis increased while fecundity exerted an even stronger positive effect on λ variation in open‐ than in shrubby‐canga (Fig. S3). To better understand the processes underlying these interannual changes, additional demographic transitions should be sampled.

#### Elasticity analysis

**Fig. 5:**
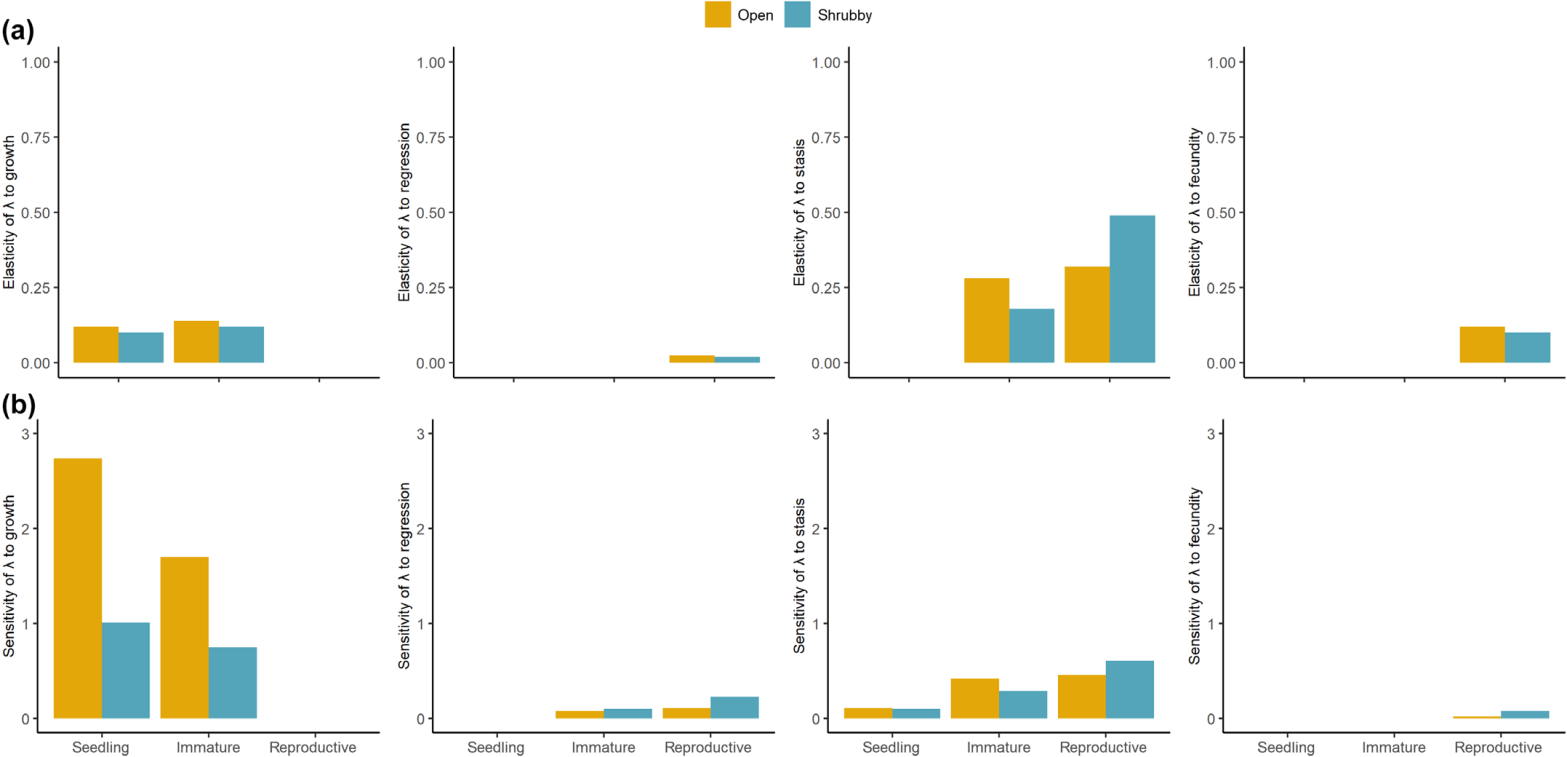
Prospective analyses of population growth in open‐ and shrubby‐canga populations of *Ipomoea cavalcantei*. Elasticity (a) and sensitivity (b) of population growth rate (λ) to upper‐level vital rates (growth, regression, stasis, and fecundity) in open‐canga (yellow) and shrubby‐canga (blue) populations. All vital rates above are conditional on survival. Elasticity and sensitivity were calculated using the mean matrix for each population.

Overall, both populations of *I. cavalcantei* exhibit similar elasticity patterns, with the highest values associated with stasis (survival and remaining in the same stage) and the lowest with growth and fecundity (Fig. 5). This indicates that potential changes in stasis have the greatest proportional effect on population growth rates. In both populations, stasis (i.e. survival) of reproductive individuals shows the highest elasticity. However, in open‐cangas, the elasticities of stasis for immature (0.28) and reproductive individuals (0.32) are similar, whereas in shrubby‐cangas, the elasticity of stasis of reproductive individuals (0.49) is much higher than that of immature individuals (0.18) (Fig. 5).

#### Plant performance of open and shrubby canga populations

Seed production (seeds m^−2^) was greater in shrubby‐cangas in comparison to open‐cangas (*P* < 0.001, Table 1), and such pattern was consistent throughout the study period (2022–2024; Fig. S4). However, in both vegetation types, the density of reproductive individuals did not differ significantly (*P* = 0.4). Thus, the higher number of seeds per m^2^ in shrubby‐canga did not result from a higher number of reproductive individuals but rather because individuals are larger with a greater number of reproductive shoots (Table 1). Conversely, the density of seedlings and immature individuals remained significantly higher in open‐cangas (Table 1; Fig. S4).

**Table 1:** Density of different ontogenetic stages (seedling, immature, and reproductive individuals), seeds produced (seeds m^−2^), number of reproductive shoots, and survival probability of *Ipomoea cavalcantei* in plots of open‐ and shrubby‐cangas.

	Open‐canga	Shrubby‐canga	*P*‐value
Density (n m^−2^)			
Seedling	0.28 ± 0.08	0.09 ± 0.02	**0.004**
Immature individuals	0.94 ± 0.2	0.28 ± 0.05	**0.005**
Reproductive individuals	0.31 ± 0.03	0.35 ± 0.02	0.4
Seed production	10 ± 2.37	23.6 ± 4.49	**< 0.001**
Reproductive shoots	0.91 ± 0.14	2.15 ± 0.24	**< 0.001**
Survival probability (%)			
Seedling	19.6 ± 5.56	32.1 ± 8.83	0.8
Immature individuals	81.4 ± 2.47	82.3 ± 4.13	0.9
Reproductive individuals	97.4 ± 1.8	98.5 ± 1.02	0.9

Densities were estimated as the mean number for all 3 yr m^−2^ (mean ± SE). Significant differences are given in bold.

Survival probability did not differ significantly between sites for any of the ontogenetic stages (Table 1). The probability of a seedling surviving the first dry season was low in both sites – *c*. 32% in shrubby‐canga and 20% in open‐canga (*P* = 0.8; Table 1). Nonetheless, the survival probability of established individuals (both immature and reproductive) was higher than 75% in both open‐ and shrubby‐cangas (Table 1).

Altogether, these results show that despite the lower seed production in open‐cangas, there was a greater number of seedlings m^−2^ with the same probability of surviving the dry season as seedlings from shrubby‐canga (Table 1). Thus, the greater fecundity in open‐cangas was due to greater seed germination and seedling emergence (i.e. greater recruitment), while slower growth hinders individuals from reaching maturity.

### Experimental response of seed germination, seed dormancy release, and seedling establishment to microclimatic conditions

#### Seed germination experiments

**Fig. 6:**
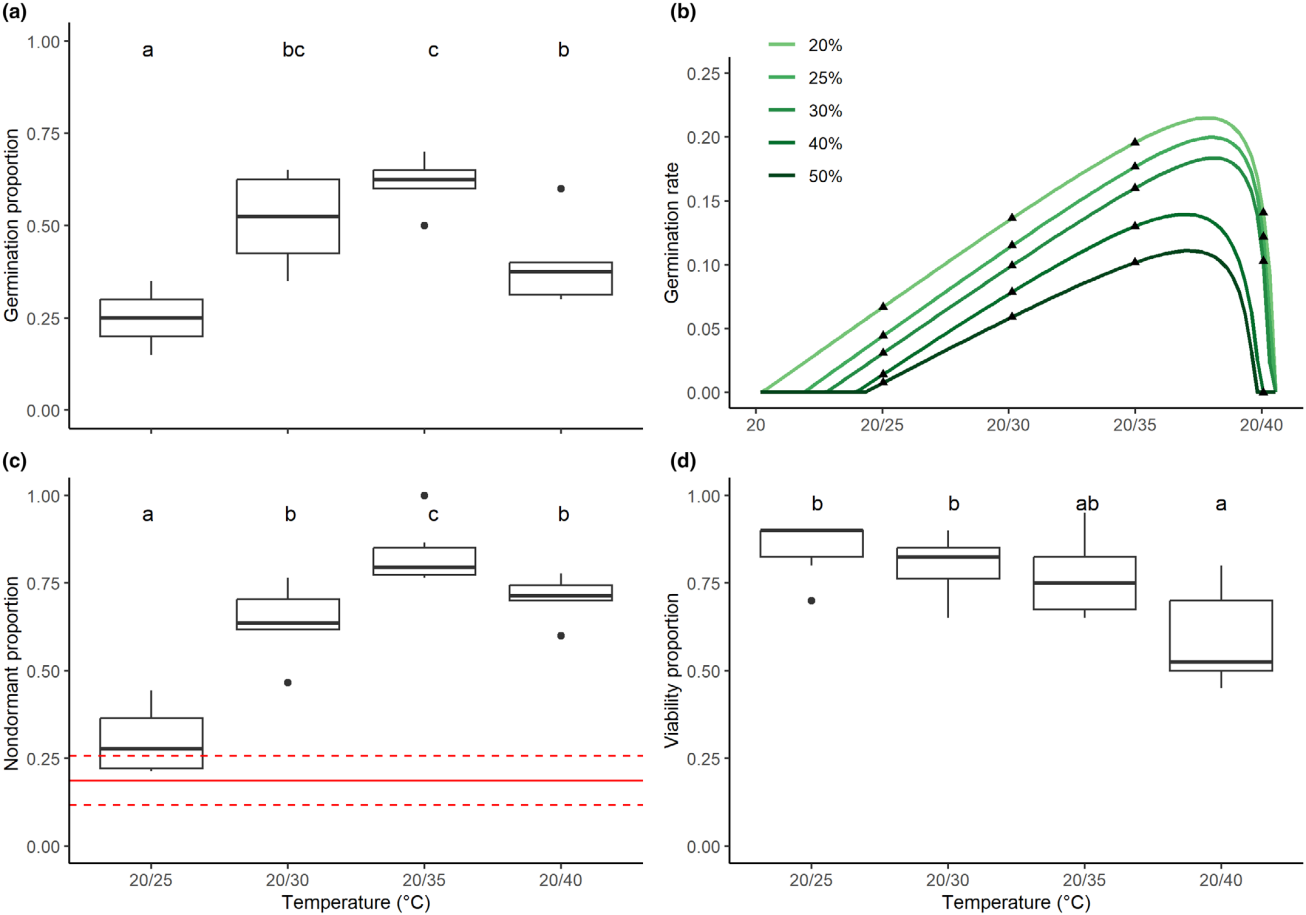
Temperature effects on seed germination responses of *Ipomoea cavalcantei*. Effect of temperature on (a) final germination proportion, (b) threshold temperatures for seed germination, (c) seed dormancy, and (d) seed viability of *Ipomoea cavalcantei*. In (b), symbols (triangles) denote the observed data, and colored lines show the hyperbolic threshold model fit to different germination percentiles. Colored lines correspond to the germination rate for the 20^th^, 25^th^, 30^th^, and 50^th^ percentiles for the entire seed lot. In (c), the proportion of seeds released from dormancy (nondormant seeds) increases with higher alternating temperatures. The red lines refer to the mean ± SD of nondormant seeds among recently dispersed seeds. Different letters indicate statistical differences (*P* < 0.05) between treatment means, as determined by Tukey's HSD *post‐hoc* test. In the boxplots, boxes represent the interquartile range (25^th^–75^th^ percentiles) with the median indicated by a horizontal line; whiskers extend to 1.5× the interquartile range; raw data points are jittered over the boxplots.

The time‐to‐event model showed a good fit to the germination data for all temperature regimens (*P*‐value < 0.001; Table S3). The germination percentage differed significantly with temperature (Likelihood ratio test permutation‐based, p‐value =0.005; 199 permutations). The greatest germination percentages were found with alternating temperatures of 20/30 and 20/35°C, while germination percentages in the lower and upper‐temperature regimens (20/25 and 20/40°C) were significantly lower (Fig. 6a).

According to the BIC values, the best model that describes the relationship between germination rate and alternating temperatures was the hyperbolic threshold model (Table S4). The model showed a good fit to the germination parameters, base and ceiling temperatures, and thermal time constant (theta) for all germination proportions (*P*‐value <0.05; Table S5). This model shows how the germination rates varied between different seed fractions (from 20 to 50% of the seeds) with the alternating temperature regimens (Fig. 6b). Base temperature for germination (mean temperature after which seeds begin germinating) varied between 20.1 and 22.2°C, which corresponds to an alternating temperature ranging from 20/21 to 20/24°C, but showed within‐lot variability and was lower for fast‐germinating seed fractions (20 and 25%; Fig. 6b). The mean ceiling temperature for germination (after which germination ceases) was close to 30°C (corresponding to alternating temperatures of 20/40°C) and appeared to be constant within each seed lot (Fig. 6b). Although the hyperbolic threshold model does not estimate the optimal germination temperature, the mean temperature close to the optimum varied between 28.4 and 29°C, corresponding to an alternating temperature of *c*. 20/38°C (Fig. 6b).

#### Dormancy release and viability

*Ipomoea cavalcantei* seeds showed physical dormancy as seed coat permeability to water remarkably increased from intact seeds (19 ± 0.07%) to manually scarified seeds (100%). Thus, most seeds (> 81%) were physically dormant when dispersed (baseline of dormancy proportion). Moreover, seed dormancy was progressively broken as temperature increased, reaching its highest dormancy release level under the alternating temperature of 20/35°C (Fig. 6c), which was the temperature used to represent the environmental condition of open‐canga vegetation (Fig. 2). Seed viability tended to decrease with the increase in temperature, being significantly lower with the 20/40°C temperature regime in relation to 20/30 and 20/25°C (Fig. 6c). That is, dormancy prevents seeds from germinating when alternating temperatures are low (< 20/30°C), while alternating temperatures higher than 20/40°C decrease seed germination as many seeds die (Fig. 6d).

#### Light effects on seedling establishment

**Fig. 7:**
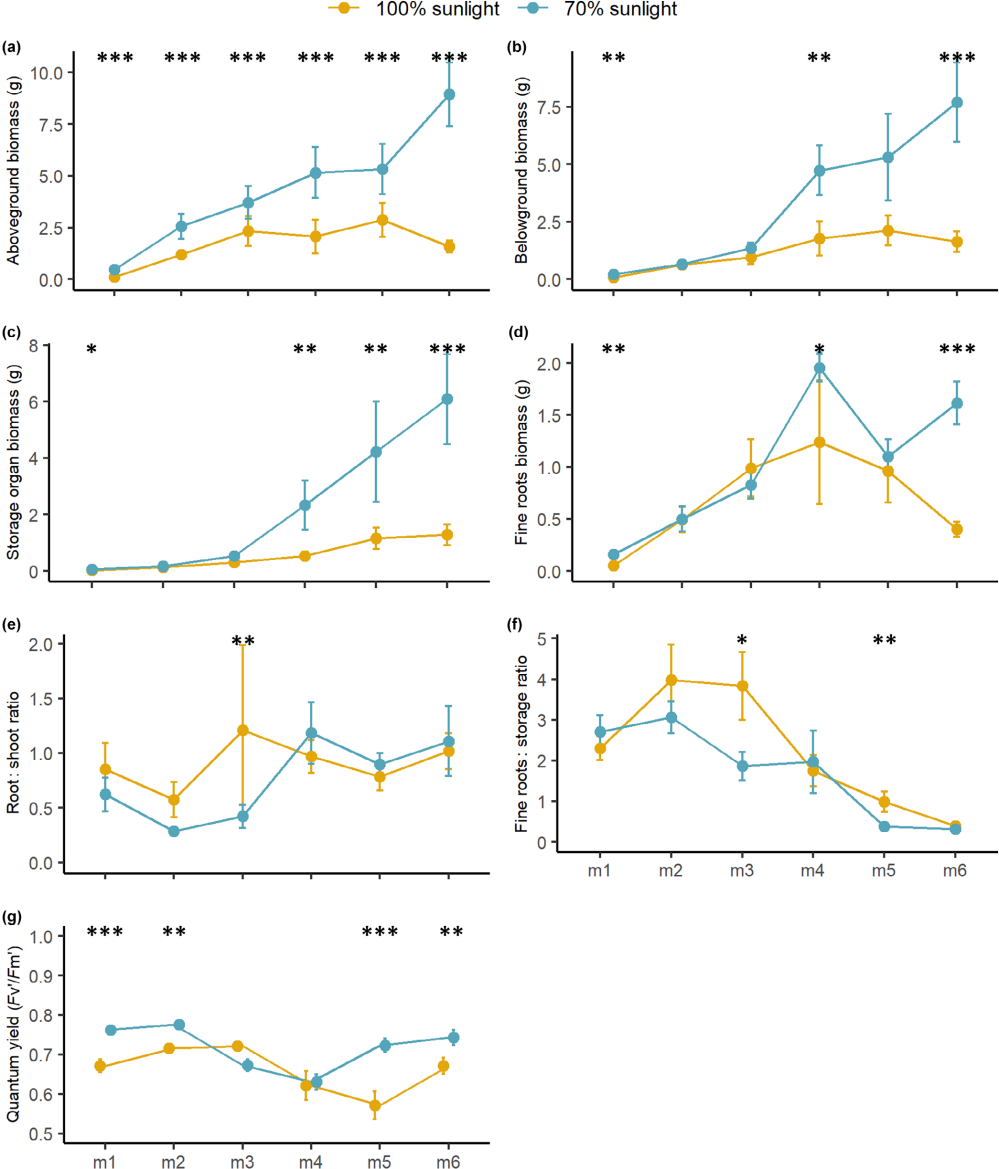
Light effects on growth and physiological responses in seedlings of *Ipomoea cavalcantei*. Biomass (dry weight) of above (a) and belowground (fine roots + storage organ; (b) parts, storage organ (c), and fine roots (d), root: shoot ratio (e), fine root: storage ratio (f), and quantum efficiency of photosystem II ($F_{v}^{\prime }/F_{m}^{\prime }$) for seedlings of *Ipomoea cavalcantei* growing for 6 months (m1–m6) under two light conditions: 100% sunlight (in yellow) and 70% sunlight (in blue). Asterisks indicate significant differences (*, *P* < 0.05; **, *P* < 0.01; ***, *P* < 0.001) and error bars represent the SE of the mean.

Above and belowground biomass was higher in plants grown under 70% sunlight (mimicking the shrubby‐canga habitat), especially after the third month when cotyledons fell off (*P* = 0.001; Fig. 7a,b). The belowground biomass was higher mainly due to the higher biomass of the storage organ (Fig. 7c), while difference in fine roots was more pronounced in the last month (Fig. 7d). However, we did not observe consistent differences in allocation of roots and shoots (root : shoot ratio; Fig. 7e) and of fine roots and storage organs (fine root : storage ratio; Fig. 7f) among plants grown under different light conditions, indicating that plants allocated resources equally to both above and belowground structures and to both absorption and storage. Additionally, the efficiency of the photosystem II was significantly lower in plants grown under full sunlight compared to those grown under 70% sunlight (Fig. 7g), indicating that growth is likely hampered by photosynthetic limitations.

## Discussion

### Demographic parameters and plant performance across vegetation types

Our hypothesis that demographic compensation could explain the ecological distribution of *Ipomoea cavalcantei* was corroborated. We found similar population performance in open‐ and shrubby‐canga despite differences in environmental conditions. However, this was driven by variation in vital rate contribution, with these rates fluctuating in opposite directions (Doak & Morris, 2010; Villellas *et al*., 2015). Although growth and fecundity had low elasticity (low impact on lambda), our life table response experiment (LTRE) showed that they were the vital rates that most contributed to the observed differences in population growth rates. Specifically, the open‐canga population exhibited a lower contribution of growth but a higher contribution of fecundity to the population growth rate, in relation to the shrubby‐canga population. Moreover, growth, particularly growth to maturity, had the highest sensitivity, while fecundity had the lowest. Nonetheless, the large differences between the two habitats (i.e. high among‐population variance) support the idea that even vital rates with low proportional contributions (low elasticity) can play a crucial role in driving demographic compensation (Santos *et al*., 2024). In addition, demographic compensation was consistent in both transitions, but was even more pronounced in the second transition, when growth and stasis showed a greater negative contribution but fecundity showed an even greater positive contribution in open‐ compared to shrubby‐canga. These results indicate that demographic compensation is a strategy that enables *I. cavalcantei* to increase the range of environments over which it occurs, as the more unfavorable environmental conditions for growth (such as the enhanced solar radiation/light incidence, higher temperatures, and reduced water availability found in open‐cangas) are buffered by compensatory changes in fecundity rates.

Fecundity can be characterized by seed production (fecundity *per se*) and number of seedlings (or seedling emergence; recruitment), which varied significantly between open‐ and shrubby‐cangas. However, since seed production is a function of plant size (Shipley & Dion, 1992), it is expected to be higher where growth is greater and plants are larger. Indeed, our results show that seed production (seed m^−2^) was higher in shrubby cangas due to the greater size of individuals (greater number of reproductive shoots) and not because of a greater number of reproductive individuals, nor because of a greater number of fruits per reproductive shoot. Since these vegetation types are not distant from each other (< 4 km), other factors determining seed production (such as the presence of pollinators; Larson & Funk, 2016) are likely to be similar. Hence, the compensation observed was actually between growth (size of individuals and, consequently, seed production) and recruitment (number of seedlings or seedling emergence), indicating that differences between populations are not driven by seed limitation but rather by different recruitment opportunities (Duncan *et al*., 2009). This also indicates that the environmental factors that differ in these vegetation types are mostly affecting growth and recruitment of *Ipomoea cavalcantei*.

Growth and recruitment have diverging environmental requirements. While environmental conditions of open‐cangas are not optimal for the growth of *I. cavalcantei*, they are favorable for recruitment, supporting the idea that the ecological niche of a species is composed of an ensemble of demographic niches in which requirements for survival, growth, and fecundity vary along the environmental axes (Grubb, 1977; Ehrlén *et al*., 2016; Pironon *et al*., 2018). Even though this has been demonstrated in several species, most studies have been carried out in temperate ecosystems with species that occur across relatively wide geographic regions where environmental variation usually implies a latitudinal (Doak & Morris, 2010; Villellas *et al*., 2013; Yang *et al*., 2022) or altitudinal gradient (García‐Camacho *et al*., 2012; Souza *et al*., 2018). Our results show that this can occur in an edaphic‐endemic species distributed across a very narrow geographic region (Giulietti *et al*., 2019), but where microclimatic heterogeneity was sufficient to affect demographic rates, increasing the number of sites the species can maintain viable, stable populations.

### Experimental response of germination and establishment to micro‐environmental conditions

Our germination and seedling establishment experiments provide mechanistic evidence for the demographic compensation observed in the field, by showing that microclimatic conditions favor recruitment in open‐cangas while constraining growth. The high recruitment observed in open‐canga vegetation can be attributed to the fact that the optimum temperature for dormancy release and germination coincided with the environmental conditions prevalent in this habitat. By contrast, the performance of seedling establishment, in terms of above and belowground biomass, was higher in plants grown under conditions resembling the shrubby‐canga vegetation, which explains the higher growth observed in the population from this vegetation type. Early life‐cycle events are strongly influenced by micro‐environmental conditions (Latimer & Jacobs, 2012; Richardson *et al*., 2012; Dayrell *et al*., 2021), such as local temperature, water availability, and light, which are critical determinants of plant recruitment processes, including seed germination, seedling emergence, and establishment (Kitajima & Fenner, 2000; Larson & Funk, 2016).

Temperature plays a crucial role in seed viability, germination and dormancy release (Alvarado & Bradford, 2002; Jayasuriya *et al*., 2008; Baskin & Baskin, 2014; Escobar *et al*., 2025), and significantly influences species distribution (Rosbakh & Poschold, 2015). Our findings indicate that the difference in seedling recruitment between habitats can be largely explained by their temperature regimes. High seed viability was maintained between 20/25 and 20/35°C, but decreased abruptly at 20/40°C. Meanwhile, seeds were gradually released from dormancy as the temperature range increased, attaining the highest dormancy release level at 20/35°C, where maximal seed germination also occurred. Therefore, dormancy likely prevents seeds from germinating in closed environments, such as forest understories and under grass tussocks, where daily temperature fluctuations are lower and the mean daily daytime (06:00–18:00 h) temperature is *c*. 25°C (Daibes *et al*., 2017). As the environment becomes more open, the likelihood of seeds germinating increases, until reaching its maximum in microhabitats with alternating temperatures near 20/35°C – a temperature regime more commonly observed in open – than shrubby‐cangas. During the germination period (i.e. the rainy season), mean daytime temperatures in open‐canga fell within the optimal range for germination and dormancy release (between 30 and 35°C) in 92 d and exceeded 30^o^C for five or more consecutive days on nine occasions. By contrast, shrubby‐cangas reached the 30–35°C range on only 43 d and surpassed 30°C for five or more consecutive days on only three occasions. Consequently, open‐canga soils provide more favorable conditions for seed germination, resulting in higher recruitment, which can be attributed to fine‐scale variation in microhabitat conditions, as reported for other canga species (Dayrell *et al*., 2021).

Other aspects of the temperature profile may also contribute to breaking dormancy and enabling germination. For example, during the rainy season, maximum temperatures frequently exceeded 50°C in open‐canga (70 d), but only occasionally in shrubby canga (23 d). Open‐cangas experienced at least 3 h above 40°C in 84 d and at least 2 h over 50°C in 42 d, whereas shrubby‐cangas experienced 3 h over 40°C in only 34 d and 2 h over 50°C in only 13 d. Greater temperature fluctuations over shorter durations have been shown to break dormancy and enable germination in physically dormant seeds of several species from fire‐prone Mediterranean ecosystems (Santana *et al*., 2013; Ooi *et al*., 2014) and tropical savannas (Daibes *et al*., 2017). These ecosystems are seasonally dry environments, and natural fires create gaps that elevate soil temperatures to levels similar to those observed in open‐cangas. Hence, further experiments exploring a wider range of temperatures and exposure duration would enhance our understanding of the temperature requirements of *I. cavalcantei* seeds. This is particularly critical for predicting the species' persistence in an ever‐hotter world (Feron *et al*., 2024), as the optimal germination temperature for *I. cavalcantei* is close to its ceiling temperature, beyond which seed mortality increases and germination ceases.

In canga ecosystems, the topographic features of the environment (e.g. soil depth, fragmented rock cover, ferricrete cover, surface inclination) create significant microclimatic variation across patches (Jacobi *et al*., 2007; Schaefer *et al*., 2016a; Mitre *et al*., 2018), ultimately shaping distinct plant communities within the same landscape (do Carmo *et al*., 2016; do Carmo & Jacobi, 2016; Gastauer *et al*., 2021). Fine‐scale variation in rock micro‐relief is associated with strategies to cope with water deficits and mechanical resistance to root growth (do Carmo *et al*., 2016; Oliveira *et al*., 2016). In open‐cangas, soil pockets rarely reach 10 cm in depth, which hinders root growth (Oliveira *et al*., 2016; Schaefer *et al*., 2016b), but soil accumulating in crevices could improve water retention and create milder environmental conditions that could favor seed recruitment (do Carmo *et al*., 2016; Dayrell *et al*., 2021). Shrubby‐cangas, on the other hand, grow on areas with more fragmented rock (Schaefer *et al*., 2016b), which allows greater root penetration and, consequently, deeper roots and larger belowground systems (Gao *et al*., 2016; Oliveira *et al*., 2016), enabling greater water uptake and storage (Lilley & Kirkegaard, 2011), but soil‐accumulated crevices do not seem to be as common (pers. observation/communication).

In addition, the deeper, looser soils of shrubby‐cangas can support larger shrub and tree species (do Carmo *et al*., 2016; Schaefer *et al*., 2016a), whose canopies create partially shaded areas with reduced light levels and temperatures at the soil level during the rainy, growing season. In these sites, the survival and growth of seedlings and juveniles should be greater than in full sunlight conditions, as extreme irradiance can negatively affect photosynthetic rates (Valladares *et al*., 2005; Pérez‐Ramos *et al*., 2013). However, despite the high irradiance endured by these plants, carbon assimilation in many *campo rupestre* species seems to be determined by stomatal limitation (water availability) and not by a reduced photosystem efficiency (Lüttge *et al*., 2007; Oliveira *et al*., 2016). Stomatal closure, deeper roots, presence of water storage tissues, and leaf‐shedding are strategies that plants use to cope with drought (Laughlin, 2023). Nonetheless, our potted experiment (under conditions where soil and water were not limiting factors) showed that plants grown under full sunlight exhibited lower root and shoot biomass as well as a lower efficiency of the photosynthetic apparatus in comparison to plants grown under milder conditions (70% light). Thus, in open‐cangas, both stomatal and photosynthetic limitations may act together, with the growth of *I. cavalcantei* constrained not only by the impenetrable bedrock, reducing access to water sources, but also by the excessive irradiance that can reduce photosynthetic efficiency.

### Conclusions

Our results highlight the relevance of investigating microclimatic drivers to better understand spatial variation in vital rates and, consequently, in population and community dynamics, particularly in heterogeneous environments. Most studies addressing climatic effects on populations focus on broad latitudinal or altitudinal gradients, whereas our study is among the few that explicitly evaluate microclimatic effects at local scales (see Blonder *et al*., 2018). Despite the strong influence of local climate conditions on species performance, species distribution models often rely on data with a coarse spatial resolution (Bennie *et al*., 2014; Compagnoni *et al*., 2021). Additionally, abiotic factors are less frequently investigated in demographic studies than biotic factors (mainly competition) or disturbance regimes (mainly fire; Ehrlén *et al*., 2016). Thus, to improve the accuracy of species distribution models, particularly when investigating environmental changes, it is crucial to examine how abiotic factors at local scales (microclimate) affect vital rates across space and time. Achieving this, however, requires long‐term demographic studies that account for microclimatic variation among populations. Although such studies can be costly and time‐consuming, they are likely to produce more robust and ecologically meaningful predictions.

In our study species, microclimatic factors affected early life‐cycle events, such as seed germination and seedling establishment, stages in which plants are at the greatest risk of mortality (Kitajima & Fenner, 2000). Although open‐cangas provide more opportunities for seed germination and seedling emergence, this did not result in a greater seedling survival and growth (i.e. in a more successful establishment). However, the increased recruitment opportunities were sufficient to ensure seedlings succeed in maintaining viable populations within this range of environmental conditions. Thus, this suggests that preserving different vegetation types may be essential to maintain a diversity of ecological strategies that allow species to persist under harsh environmental conditions, particularly in the face of ongoing environmental changes (Bijlsma & Loeschcke, 2012; Snell *et al*., 2019).

## Competing interests

The authors declare the following financial interests/personal relationships, which may be considered as potential competing interests: all authors report that financial support was provided by Vale.

## Author contributions

TZ, DFE, VAK, RCQP, CFC, CSC contributed to study conception and experimental design. DFE, VAK, RCQP, CFC, CSC. TZ, DFE, VAK, GSS, YND, MW, RLA, RCQP and CSC contributed to data collection. TZ, DFE, GSS and CSC contributed to data collection, analysis and interpretation. TZ contributed to manuscript preparation (first draft). TZ, DFE, GSS, RLA, RCQP, VT and CSC contributed to the review and editing. TZ and DFE contributed equally to this work.

## Disclaimer

The New Phytologist Foundation remains neutral with regard to jurisdictional claims in maps and in any institutional affiliations.

## Supporting information


**Fig. S1** Upper‐level vital rates of *Ipomoea cavalcantei* for each annual transition in open‐ and shrubby‐canga populations.
**Fig. S2** Lower‐level vital rates of *Ipomoea cavalcantei* for each annual transition in open‐ and shrubby‐canga populations.
**Fig. S3** Vital rate contributions to the observed differences in population growth rates (Δλ) between the open‐ and shrubby‐canga populations of *Ipomoea cavalcantei* for each transition.
**Fig. S4** Reproductive output and population structure of *Ipomoea cavalcantei*.
**Methods S1** Methods to determine demographic parameters, seed germination and dormancy response to alternating temperature, and seedling growth to light conditions of *Ipomoea cavalcantei* populations in Amazon canga.
**Table S1** Definitions and calculation procedures for all seed dormancy, imbibition, germination, and viability measures used in the study.
**Table S2** Temperature and PPFD (Photosynthetic Photon Flux Density) recorded in shrubby‐ and open‐canga areas during the rainy season.
**Table S3** Logistic model of germination for *Ipomoea cavalcantei* in response to alternating temperature treatments.
**Table S4** Competing threshold thermal time models tested to evaluate the relationship between alternating temperatures and germination rate for *Ipomoea cavalcantei*.
**Table S5** Results of the hyperbolic threshold model.
Please note: Wiley is not responsible for the content or functionality of any Supporting Information supplied by the authors. Any queries (other than missing material) should be directed to the *New Phytologist* Central Office.

## Data Availability

The data and source code are stored in FigShare at doi: 10.6084/m9.figshare.30267631.
