# Supplementary material for: Microclimate drives demographic compensation in a narrow endemic tropical species
Source: New Phytol. 2026 Feb 11;250(1):166–80. doi: 10.1111/nph.70944 (PMC12961258; doi:10.1111/nph.70944)
Supplement: Supplementary file 1 — Fig. S1 Upper‐level vital rates of Ipomoea cavalcantei for each annual transition in open‐ and shrubby‐canga populations. Fig. S2 Lower‐level vital rates of Ipomoea cavalcantei for each annual transition in open‐ and shrubby‐canga populations. Fig. S3 Vital rate contributions to the observed differences in population growth rates (Δλ) between the open‐ and shrubby‐canga populations of Ipomoea cavalcantei for each transition. Fig. S4 Reproductive output and population structure of Ipomoea cavalcantei. Methods S1 Methods to determine demographic parameters, seed germination and dormancy response to alternating temperature, and seedling growth to light conditions of Ipomoea cavalcantei populations in Amazon canga. Table S1 Definitions and calculation procedures for all seed dormancy, imbibition, germination, and viability measures used in the study. Table S2 Temperature and PPFD (Photosynthetic Photon Flux Density) recorded in shrubby‐ and open‐canga areas during the rainy season. Table S3 Logistic model of germination for Ipomoea cavalcantei in response to alternating temperature treatments. Table S4 Competing threshold thermal time models tested to evaluate the relationship between alternating temperatures and germination rate for Ipomoea cavalcantei. Table S5 Results of the hyperbolic threshold model. Please note: Wiley is not responsible for the content or functionality of any Supporting Information supplied by the authors. Any queries (other than missing material) should be directed to the New Phytologist Central Office. [file NPH-250-166-s001.pdf]

New Phytologist Supporting Information

Article title: Microclimate drives demographic compensation in a narrow endemic tropical species

Authors: Talita Zupo (Orcid ID: 0000-0001-5873-542X) and Diego Fernando Escobar (Orcid ID: 0000-0002-5091-3006), Gabriel S. Santos (Orcid ID: 0000-0001-7991-8807) , Vitor de Andrade Kamimura (Orcid ID: 0000-0002-3276-5812), Yan Nunes Dias (Orcid ID: 0000-0002-4058-9433), Rafael L. de Assis (Orcid ID: 0000-0001-8468-6414), Cecílio F. Caldeira (Orcid ID: 0000-0003-4762-3515), Maurício Watanabe (Orcid ID: 0000-0001-9690-5565), Rita de Cássia Quitete Portela (Orcid ID: 0000-0002-8702-7665), Valeria Tavares (Orcid ID: 0000-0003-0966-0139), Carolina da Silva Carvalho (Orcid ID: 0000-0002-0063-2185)

Article acceptance date: 18 December 2025

Methods S1

Estimation of seed predation prior to dispersion

We harvested more than 300 fruits to obtain the necessary number of viable seeds for our germination experiments. Each fruit can contain up to 4 viable seeds, but many seeds are predated or infested with fungi. Therefore, sorting of seeds is essential prior to the experiments. While sorting the seeds, we quantified the proportion of predated and infected seeds. To do so, we separated two sets of 100 fruits and counted the amount of viable (visually intact) and non-viable seeds. Nearly 70% of seeds were considered non-viable due to predation or fungal infestation.

Construction of projection matrices

The projection matrix is composed of matrix elements (a_{ij}) that represent the transition probabilities or fecundity rates, describing how stage *j* at time *t* contributes to stage *i* at time *t + 1* (Caswell 2001). For *Ipomoea cavalcantei*, the matrix elements (or upper-level vital rates) represent stasis (S_{ij}), regression (R_{ij}), growth (G_{ij}), and fecundity (F_{ij}), and are estimated as a function of lower-level vital rates (survival [s_i], growth [$g_{i,}$], and reproduction [$f_{i,}$]). We assume that *I. cavalcantei* forms a transient seed bank given it is a perennial, iteroparous species with vegetative regeneration submitted to a seasonally predictable climate (Gioria et al., 2020). Additionally, soil seed banks in

campo rupestre seem to have low seed density and diversity (Medina & Fernandes 2007), particularly of woody species (Luz, 2018), indicating that regeneration via seeds is more likely to occur from recently dispersed seeds. Thus, we considered that seeds produced in time *t* germinate or die in time *t +1*. For all matrix analyses, we used the "popbio" package (Stubben and Milligan, 2007) in R (R Development Core Team, 2024).

Seed germination experiments

We conducted germination experiments under different alternating temperatures to evaluate the effect of temperature on seed germination of *I. cavalcantei.* Also, this allowed us to understand if soil temperatures act as a filter for seed germination, potentially driving differences in plant performance between open- and shrubby-cangas. For such, seeds from over 50 *I. cavalcantei* individuals were harvested in May 2022, along both *canga* vegetation types. Seeds of both vegetation types were pooled, sorted, and put to germinate under four different alternating temperatures (night/day: 20/25, 20/30, 20/35, and 20/40 °C; 12h under each temperature) in a plant growth chamber (Fitotron SGC120, Weiss Technik, United Kingdom). The photoperiod was set at 12 h light (50 micromol.m^{-2}.s^{-1}) at the highest temperature and 12 h dark at the lowest temperature. For each temperature, we used six replicates with 20 seeds each (120 seeds/treatment), where seeds were placed in 9 cm Petri dishes with two layers of filter paper saturated with deionized water. The time between seed harvest and the start of the germination experiments was less than four weeks.

The temperature treatments were based on soil temperatures measured during the rainy season in open- and shrubby-cangas, which exhibit different daily temperature cycles (20/35 and 20/30 °C, respectively). Although the mean daily temperatures differed only slightly between habitats (28 ± 2.3 °C in open-cangas and 26.1 ± 1.8 °C in shrubby-cangas), these correspond to the 20/35 (27.5 °C) and 20/30 (25 °C) daily regimens. We also included cooler (20/25 °C) and warmer (20/40 °C) daily cycles to broaden the range of diurnal fluctuations (5–20 °C) and mean temperatures (22.5–30 °C) observed across canga vegetation types.

Seed germination was recorded three times a week for 20 days, and germination was determined by radicle emergence (Bewley et al., 2013). At the end of the experiments, seeds that were covered with fungi and collapsed when pinched were recorded as dead, swollen non-germinated seeds were submitted to tetrazolium test (1 % solution at 38 °C for 72 h) to access seed viability; hard non-germinated seeds were

manually scarified with a sandpaper and allowed to germinate for one week in their original temperature and light regime. Within this period, the germinated seeds were counted as viable and dormant at the end of the germination assay; the remaining non-germinated seeds were all rotten and then classified as dead.

We determined the threshold temperatures for seed germination in two steps. First, we fitted time-to-event curves separately for each temperature regime to estimate germination dynamics, accounting for both time and proportion of germination. From each curve, we then estimated the final germination proportion and the germinate rate percentiles and tested whether they differed across temperature regimes. Time-to-event curves were fitted separately for each temperature regime to avoid degenerate curves and convergence errors that can occur when germination is very rapid at certain temperatures, which prevents us from observing a complete time course. Accurate curves are essential because threshold models rely on germination parameters derived from each curve (see Onofri et al., 2018). The time-to-event curves were fitted using the *drm* function in the *drc* package in R software, assuming a log-normal distribution for germination time (Ritz et al., 2015). The interval-censoring was estimated with the interval between the germination census rather than the census day as the independent variable (Onofri et al., 2018). The upper limit to seed germination was set to 90% as this was the maximum viability of the seed lot. We chose to set that limit for all treatments rather than correcting seed germination by seed viability of each temperature because seed viability tended to decrease as the temperature increased and this can overestimate seed germination at the highest temperatures. The fitted germination curves, expressed as a cumulative distribution function (CDF), were compared using grouped-permutation likelihood ratio tests of the *compCDF* function in the *drcte* package in R (Onofri et al., 2018); the Petri dishes were set as cluster units and 199 permutations were carried out. The final germination proportions were compared using the function *compParm,* a function used for post-hoc comparisons, implemented in the *drc* package in R software (Ritz et al., 2015).

We used the previously estimated germination rate for the 20th, 25th, 30th, 40th, and 50th percentiles to describe the relationship between germination rates and temperature using several threshold thermal-time models. Such percentiles capture the core range of germination from faster-germinating seeds to the median of the population, providing a robust representation of how the majority of seeds respond to different temperature regimes. The germination rate was estimated using the average daily

temperature (average of the two temperatures in the interval) based on Masin et al. (2017). We then fitted all threshold models available within the *drcSeedGerm* package in R software that estimates the base and ceiling temperatures (Onofri et al., 2018). This package includes models that assume a linear (*e.g.,* broken-stick model) or curvilinear (*e.g.,* exponential-type models) relationship between germination rate and temperature. Models were compared using the Bayesian information criterion (Schwarz, 1978). For more details on thermal models see Onofri et al. (2018).

Dormancy release and viability

Although our demographic model assumed that seeds either germinate or die within a year (i.e., transient soil seed banks), many Convolvulaceae produce physically dormant seeds (Jayasuriya et al. 2008). If seeds remain viable but ungerminated across multiple years (i.e., persistent soil seed banks), this could buffer populations against temporal variability in recruitment and influence population dynamics. In addition, seed dormancy can regulate the spatial distribution of germination, as the cues for dormancy release enables germination to occur under optimal conditions for establishment (Donohue et al., 2010). We therefore performed dormancy release experiments to test whether seeds exhibit physical dormancy and to assess the conditions required for its release. By linking seed dormancy to germination patterns, these experiments provide critical context to interpret recruitment in the field and to validate our assumption of a transient soil seed bank for this species.

To access seed dormancy release and viability, firstly, we determined the seed coat permeability to water by comparing imbibition between intact seeds (no pre-treatment) with mechanically scarified seeds (30 seeds/treatment). For such, seeds were weighed individually and then incubated at room temperature (ca., 23 °C) and 12/12 h white light/darkness in germination boxes. Seeds were placed over two layers of filter paper saturated with distilled water and covered with another layer of filter paper saturated with distilled water. After seven days of incubation, seeds were weighed again, individually, and the percentage increase in fresh weight (P%) for each seed was calculated with the formula, $P\% = [(P2 - P1)/P1] \times 100$, where P1 and P2 represent the fresh weights at times t1 and t2, respectively (Paiva et al. 2006; Orozco-Segovia et al. 2007). Non-imbibed intact seeds at the end of the experiment were classified as dormant, while imbibed seeds were classified as non-dormant.

Then, we evaluated if and which alternating temperature could break physical seed dormancy by comparing the proportion of seed dormancy and viability after undergoing different alternating temperature regimens *(seed germination experiments*). Germinated and swollen seeds that did not germinate at the end of the germination experiment were classified as non-dormant, whereas hard non-germinated seeds were classified as dormant. Seed viability was calculated as the sum of germinated seeds, seeds considered viable after the tetrazoluim tests, and seeds that germinated after manual scarification. We determined the proportion of seed dormancy based on viable seeds at the end of the experiment rather than all 20 seeds because high alternating temperatures can act both breaking seed dormancy or killing the seeds. Moreover, the maximum seed lot viability was estimated as the highest viability per Petri dish at the 20/25°C temperature regime, given higher temperatures could be lethal to seeds.

Finally, we tested the effect of alternating temperature regimes on seed dormancy release and seed viability by fitting two generalized linear models with binomial errors and logit link function. For seed dormancy release, non-dormant seeds at the end of the germination experiment were codified as (1) and dormant ones as (0), whereas for seed viability, viable seeds were codified as (1) and dead seeds as (0). The generalized linear models (GLM) were fitted using the *glm* function in R software (R Core Development Team 2024). Robust standard error was estimated with the clustered covariance matrix method using the Petri dish as the cluster unit, using the *coeftest* function from the *lmtest* package and the *vcovCL* function from the *sandwich* package (Zeileis, 2006).

Supplementary Table S1. Definitions and calculation procedures for all seed dormancy, imbibition, germination, and viability measures used in the study.

Variable	Experiment	Purpose/ interpretation	Definition
Dormancy (seed coat permeability)	Seed permeability/ water imbibition test (30 seeds/treatment)	Compares seed permeability of intact and scarified seeds. Provides estimate of number of seeds with physical dormancy at dispersal.	Dormant = seeds that did not imbibe water; Non-dormant = seeds that imbibed
Dormancy release (across temperature treatments)	Germination under alternating temperatures (20 seeds/replicate. 6 replicates/treatment)	Estimates dormancy release after temperature treatments. Only viable seeds were used to avoid counting seeds killed by high temperatures as "dormant."	Dormant = hard, non-germinated seeds at the end of the trial; Non-dormant = germinated seeds + swollen (imbibed but not germinated) seeds
Seed viability (across temperature treatments)	Germination (same as above) + TZ test + post-scarification test (20 seeds/replicate. Six replicates/treatment)	Captures full viability even when dormancy prevents germination in the temperature treatment.	Viable = (1) germinated seeds + (2) seeds that germinated after manual scarification + (3) seeds staining positive in tetrazolium
Maximum seed lot viability	Maximum viability at 20/25°C (observed across the six replicates)	Used as reference because higher temperatures may induce mortality; avoids underestimating the seed lot's true viability.	Maximum viability recorded at the 20/25°C treatment

Seedling establishment experiment

Seedlings from the germination experiment were sown in 1 dm^3 pots filled with soil from a secondary forest area (Yellow Oxisol, previously fertilized) and Carolina soil® (in a 9:1 ratio) and set to grow in a greenhouse under the two light conditions established; a total of 84 seedlings were sown (42 in each treatment). Pots were irrigated to field capacity twice a day and seedlings grew in similar soil conditions to better isolate the effects of light incidence on seedling growth.

Supplementary Table S2. Detailed information on temperature and PPFD (Photosynthetic Photon Flux Density) recorded in shrubby- and open-canga areas during the rainy season (November to April). Daytime: 6 a.m. to 6 p.m.; nighttime: 6 p.m. to 6 a.m.

	**Shrubby-canga**	**Open-canga**
Mean daily temperature (day + night) + standard error	26.1 ± 1.8 °C	28 ± 2.3 °C
Mean daily daytime temperature + standard error	28.6 ± 7.3 °C	31.4 ± 9.4 °C
Mean daily nighttime temperature + standard error	22.9 ± 1.67 °C	23.4 ± 1.82 °C
Number of days in which mean daily temperature reached between 30 – 35 ºC	48 days	92 days
Number of occasions in which mean daily temperature reached over 30 ºC for five or more consecutive days	3 occasions	9 occasions
Number of days maximum temperature exceeded 40 ºC	96 days	128 days
Number of days maximum temperature exceeded 50 ºC	23 days	70 days
Number of days maximum temperature exceeded 60 ºC	1 day	5 days
Mean daily daytime PPFD + standard error	205 ± 30.4 µmol	328 ± 48 µmol
Absolute maximum daytime PPFD	1823 µmol	2276 µmol

Supplementary Figure S1: Upper-level vital rates for each annual transition in both populations of *Ipomoea cavalcantei*. The letter O stands for open-canga and S for shrubby-canga. The first transition is coded as 1 and the second transition is coded as 2.

$$O1 = \begin{pmatrix} 0 & 0 & 2.79 \\ 0.26 & 0.84 & 0.29 \\ 0 & 0.05 & 0.71 \end{pmatrix} \qquad O2 = \begin{pmatrix} 0 & 0 & 7.71 \\ 0.2 & 0.65 & 0.17 \\ 0 & 0.13 & 0.83 \end{pmatrix}$$

$$S1 = \begin{pmatrix} 0 & 0 & 1.27 \\ 0.4 & 0.75 & 0.14 \\ 0 & 0.14 & 0.85 \end{pmatrix} \qquad S2 = \begin{pmatrix} 0 & 0 & 1.42 \\ 0.38 & 0.6 & 0.\ 02 \\ 0 & 0.21 & 0.97 \end{pmatrix}$$

Supplementary Figure S2: Lower-level vital rates for each annual transition in both populations of *Ipomoea cavalcantei*. The letter O stands for open-canga and S for shrubby-canga. The first transition is coded as 1 and the second transition is coded as 2.

$$o1 = \begin{pmatrix} 0 & 0 & 1*2.79 \\ 0.26*1 & 0.88*0.94 & 1*0.29 \\ 0 & 0.88*0.06 & 1*0.71 \end{pmatrix} \qquad o2 = \begin{pmatrix} 0 & 0 & 1*7.71 \\ 0.2*1 & 0.78*0.83 & 1*0.17 \\ 0 & 0.78*0.17 & 1*0.83 \end{pmatrix}$$

$$s1 = \begin{pmatrix} 0 & 0 & 0.98*1.29 \\ 0.4*1 & 0.88*0.85 & 0.99*0.14 \\ 0 & 0.88*0.15 & 0.99*0.86 \end{pmatrix} \qquad s2 = \begin{pmatrix} 0 & 0 & 0.98*1.45 \\ 0.38*1 & 0.81*0.74 & 0.98*0.02 \\ 0 & 0.81*0.26 & 0.98*0.98 \end{pmatrix}$$

Supplementary Figure S3: Vital rate (growth, regression, stasis, and fecundity) contributions to the observed differences in population growth rates (**Δ**λ) between the open- and shrubby-canga populations of *Ipomoea cavalcantei* for each transition. Year 1 refers to the 2022-2023 transition and Year 2 to the 2023-2024 transition.

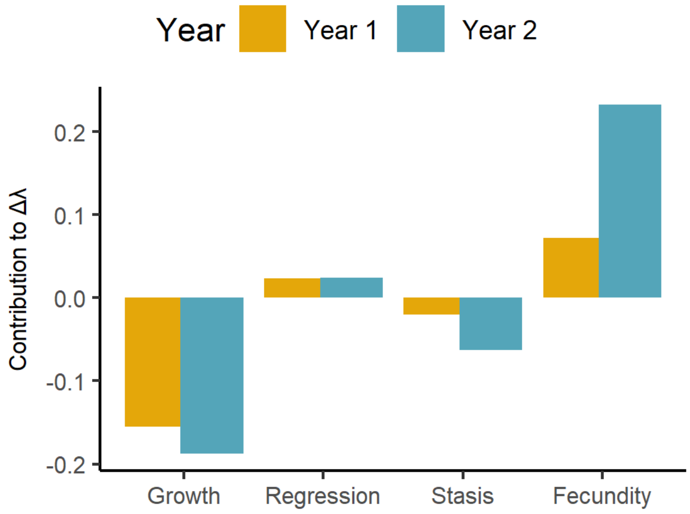

Supplementary Figure S4: Reproductive output and population structure of *Ipomoea cavalcantei*. Seed production (A) and densities (individuals m^{-2}) of seedlings (B), immature (C), and reproductive (B) plants in plots of both vegetation types for each sampled year. In the boxplots, boxes represent the interquartile range (25th–75th percentiles) with the median indicated by a horizontal line, whiskers extend to 1.5 × the interquartile range; raw data points are jittered over the boxplots.

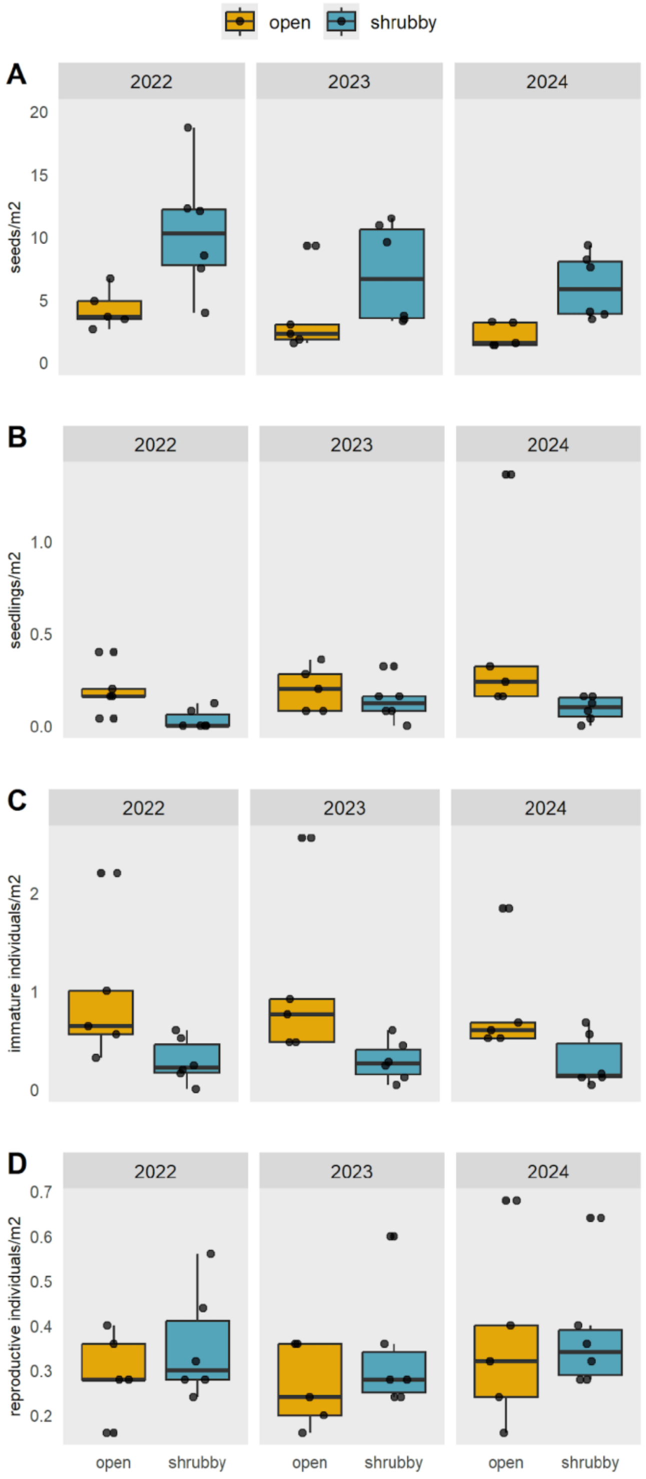

Supplementary Table S3. Logistic model of germination for *Ipomoea cavalcantei* in response to alternating temperature treatments with a maximum germination of 90%.

Temperature regimen	Mean germination proportion	Std. Error	t-value	p-value
20/25 ° C	0.243	0.027	8.867	**<0.001**
20/30 ° C	0.695	0.128	5.413	**<0.001**
20/35 ° C	0.634	0.046	13.623	**<0.001**
20/40 ° C	0.396	0.045	8.772	**<0.001**

* A p-value < 0.05 indicates that the data fit the model.

Supplementary Table S4. Competing threshold thermal time models tested to evaluate the relationship between alternating temperatures and germination rate for *Ipomoea cavalcantei.*

Model	Df	BIC
Exponential switch-off	21	-51.952
Hyperbolic	21	**-70.967**
Broken-stick	21	30.71
Polynomial	16	15.731
Broken curvilinear	21	-43.494

* The best model is shown in bold.

Supplementary Table S5. Results of the hyperbolic threshold model to evaluate the relationship between alternating temperatures and germination rate for *Ipomoea cavalcantei*.

Parameter	Estimate	Std. Error	z value	Pr(>|z|)
k:20%	0.02347	10	0.002347	0.998127
k:25%	0.021691	10	0.002169	0.998269
k:30%	0.021392	10	0.002139	0.998293
k:40%	0.052024	10	0.005202	0.995849
k:50%	0.04116	10	0.004116	0.996716
Tb:20%	20.1198	10	2.01198	**0.044222**
Tb:25%	20.94369	10	2.094369	**0.036227**
Tb:30%	21.38714	10	2.138714	**0.032459**
Tb:40%	21.97346	10	2.197346	**0.027996**
Tb:50%	22.15043	10	2.215043	**0.026757**
Tc:20%	30.2664	10	3.02664	**0.002473**
Tc:25%	30.20624	10	3.020624	**0.002523**
Tc:30%	30.17125	10	3.017125	**0.002552**
Tc:40%	29.96648	10	2.996648	**0.00273**
Tc:50%	29.86674	10	2.986674	**0.00282**
ThetaT:20%	35.47103	10	3.547103	**0.000389**
ThetaT:25%	35.21494	10	3.521494	**0.000429**
ThetaT:30%	36.38279	10	3.638279	**0.000274**
ThetaT:40%	37.99978	10	3.799978	**0.000145**
ThetaT:50%	48.02788	10	4.802788	**1.56E-06**

* Well-adjusted parameters of the hyperbolic threshold model are shown in bold.

References

Bewley, J.D., Bradford, K.J., Hilhorst, H.W.M., Nonogaki, H., 2013. Seeds: Physiology of Development, Germination and Dormancy, 3rd Edition. Springer, New York, NY. https://doi.org/10.1007/978-1-4614-4693-4

Caswell, H., 2001. Matrix population models. Sunderland; Sinauer Associates; 2. ed; 2001. 722 p.

Donohue, K., Casas, R.R. de, Burghardt, L., Kovach, K., Willis, C.G., 2010. Germination, Postgermination Adaptation, and Species Ecological Ranges. Annual Review of Ecology, Evolution, and Systematics 41, 293–319. https://doi.org/10.1146/annurev-ecolsys-102209-144715Gioria, M., Pyšek, P., Baskin, C.C., Carta, A., 2020. Phylogenetic relatedness mediates persistence and density of soil seed banks. Journal of Ecology 108, 2121–2131. https://doi.org/10.1111/1365-2745.13437

Jayasuriya, K.M.G.G., Baskin, J.M., Baskin, C.C., 2008. Dormancy, germination requirements and storage behaviour of seeds of Convolvulaceae (Solanales) and evolutionary considerations. Seed Sci. Res. 18, 223–237. https://doi.org/10.1017/S0960258508094750

Luz, G.R. da, 2018. Ecologia do banco de sementes do solo de campos rupestres: efeito da altitude, do solo, do clima e do fogo. https://hdl.handle.net/1843/36167

Medina, M.B.O., Fernandes, G.W. 2007. The potential of natural regeneration of rocky outcrop vegetation on rupestrian field soils in Serra do Cipó, Brazil. Rev Bras Bot 30: 665–678

Masin, R., Onofri, A., Gasparini, V., Zanin, G., 2017. Can alternating temperatures be used to estimate base temperature for seed germination? Weed Research 57, 390–398. https://doi.org/10.1111/wre.12270

Onofri, A., Benincasa, P., Mesgaran, M.B., Ritz, C., 2018. Hydrothermal-time-to-event models for seed germination. European Journal of Agronomy 101, 129–139. https://doi.org/10.1016/j.eja.2018.08.011

Orozco-Segovia, A., J. Márquez-Guzmán, M. E. Sánchez-Coronado, A. Gamboa de Buen, J. M. Baskin, e C. C. Baskin. 2007. “Seed Anatomy and Water Uptake in Relation to Seed Dormancy in Opuntia tomentosa (Cactaceae, Opuntioideae)”. *Annals of Botany* 99 (4): 581–92. https://doi.org/10.1093/aob/mcm001.

Paiva, EAS, Lemos-Filho, JP, Oliveira, DMT. 2006. "Imbibition of Swietenia macrophylla (Meliaceae) Seeds: The Role of Stomata". *Annals of Botany* 98 (1): 213–17. https://doi.org/10.1093/aob/mcl090.

Ritz, C., Baty, F., Streibig, J.C., Gerhard, D., 2015. Dose-Response Analysis Using R. PLOS ONE 10, e0146021. https://doi.org/10.1371/journal.pone.0146021

Schwarz, G., 1978. Estimating the Dimension of a Model. The Annals of Statistics 6, 461–464. https://doi.org/10.1214/aos/1176344136

Stubben, C., Milligan, B., 2007. Estimating and Analyzing Demographic Models Using the popbio Package in R. Journal of Statistical Software 22, 1–23. https://doi.org/10.18637/jss.v022.i11

Zeileis, A., 2006. Object-oriented Computation of Sandwich Estimators. Journal of Statistical Software 16, 1–16. https://doi.org/10.18637/jss.v016.i09
